# Three Divergent Subpopulations of the Malaria Parasite *Plasmodium knowlesi*

**DOI:** 10.3201/eid2304.161738

**Published:** 2017-04

**Authors:** Paul C.S. Divis, Lee C. Lin, Jeffrine J. Rovie-Ryan, Khamisah A. Kadir, Fread Anderios, Shamilah Hisam, Reuben S.K. Sharma, Balbir Singh, David J. Conway

**Affiliations:** Malaria Research Centre, Universiti Malaysia Sarawak, Kota Samarahan, Sarawak, Malaysia (P.C.S. Divis, K.A. Kadir, B. Singh, D.J. Conway);; London School of Hygiene and Tropical Medicine, London, United Kingdom (P.C.S. Divis, D.J. Conway);; Universiti Putra Malaysia, Serdang, Malaysia (L.C. Lin, R.S.K. Sharma);; Department of Wildlife and National Parks Peninsular Malaysia, Kuala Lumpur, Malaysia (J.J. Rovie-Ryan);; Sabah State Public Health Laboratory, Kota Kinabalu, Malaysia (F. Anderios);; Institute for Medical Research, Kuala Lumpur (S. Hisam)

**Keywords:** *Plasmodium knowlesi*, reservoir hosts, microsatellite genotypes, geographical divergence, Malaysia, parasites, macaques, zoonoses, malaria

## Abstract

Multilocus microsatellite genotyping of *Plasmodium knowlesi* isolates previously indicated 2 divergent parasite subpopulations in humans on the island of Borneo, each associated with a different macaque reservoir host species. Geographic divergence was also apparent, and independent sequence data have indicated particularly deep divergence between parasites from mainland Southeast Asia and Borneo. To resolve the overall population structure, multilocus microsatellite genotyping was conducted on a new sample of 182 *P. knowlesi* infections (obtained from 134 humans and 48 wild macaques) from diverse areas of Malaysia, first analyzed separately and then in combination with previous data. All analyses confirmed 2 divergent clusters of human cases in Malaysian Borneo, associated with long-tailed macaques and pig-tailed macaques, and a third cluster in humans and most macaques in peninsular Malaysia. High levels of pairwise divergence between each of these sympatric and allopatric subpopulations have implications for the epidemiology and control of this zoonotic species.

*Plasmodium knowlesi* is a zoonotic malaria parasite that has only recently been recognized as a notable cause of malaria (*1*). Although cases have now been seen in most countries in Southeast Asia, the largest numbers have been reported in Malaysia (*1**–**4*). The extent to which this is a result of varying efforts in diagnosis is unclear, as specific molecular identification is required to discriminate *P. knowlesi* from other malaria parasite species. Moreover, although most reports are of cases presenting with clinical symptoms, asymptomatic infections may also occur (*5*).

The *Plasmodium knowlesi* parasite is transmitted by mosquitoes to humans from monkey reservoir hosts, with different *Anopheles* species of the Leucosphyrus group having been incriminated as potential vectors in different areas (*1*,*6*). Two macaque species, the long-tailed macaque (*Macaca fascicularis*) and the pig-tailed macaque (*M*. *nemestrina*), are the major reservoirs of infection (*7*,*8*). Human infections in Malaysian Borneo, the portion of Malaysia on the island of Borneo, have divergent genetic subpopulations that are seen in the different macaque species locally, indicating that 2 independent zoonoses may be occurring sympatrically (*9*). Noticeable geographic differentiation of parasites between Malaysian Borneo and peninsular Malaysia was also evident in microsatellite analysis; separate studies have revealed divergence between the 2 regions at unlinked genes encoding the normocyte binding protein (*10*–*12*) and the Duffy binding protein (*13*,*14*), as well as the 18S rRNA and mitochondrial cytochrome oxidase subunit 1 (*15*). Whole-genome sequencing has confirmed the presence of 2 divergent subpopulations of *P. knowlesi* in Malaysian Borneo and revealed a third divergent cluster of laboratory isolates maintained in laboratories since the 1960s; most of these were recorded to have originated from peninsular Malaysia (*16*).

To resolve the population structure in relation to host species and geography, a new collection of 182 *P. knowlesi* infection samples from humans and wild macaques living in diverse areas of Malaysia was genotyped at 10 microsatellite loci. We first analyzed the new dataset separately and then analyzed a combined dataset incorporating previous multilocus microsatellite data, using several independent and complementary statistical approaches to identify genetic substructure. All analyses revealed that 2 divergent genetic subpopulations of human cases occur sympatrically in Malaysian Borneo, detected separately in long-tailed macaques and pig-tailed macaques in the same region, whereas a third divergent genetic subpopulation occurs in humans and most macaques in peninsular Malaysia. This parasite species has undergone different sympatric and allopatric processes of divergence, which will affect its future adaptation to a changing environmental landscape. Current differences between the subpopulations need to be recognized in clinical and epidemiologic studies.

## Materials and Methods

### Study Sites and DNA Samples

**Figure 1:**
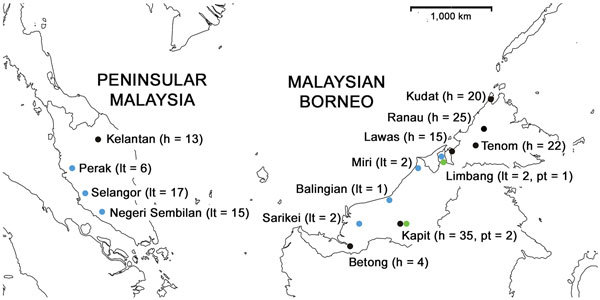
Geographic distribution of DNA samples of *Plasmodium knowlesi* infections derived from 134 humans and 48 macaques across Malaysia. h, human samples; lt, long-tailed macaque samples; pt, pig-tailed macaque samples.

We obtained blood samples infected with *P. knowlesi* from human clinical cases at 7 sites and from macaque hosts at 8 sites across Malaysia (Figure 1). We extracted DNA from anticoagulated venous blood samples or dried blood spots, and tested the DNA for the presence of different malaria parasite species by species-specific PCR using methods described previously (*7*). Samples from 134 *P. knowlesi*–positive human cases collected during 2012–2014 that had sufficient DNA for multilocus genotyping originated from Kapit (n = 35), Betong (n = 4), and Lawas (n = 15) in Sarawak state, Malaysian Borneo; from Kudat (n = 20), Ranau (n = 25), and Tenom (n = 22) in Sabah state, Malaysian Borneo; and from Kelantan (n = 13) in peninsular Malaysia.

Samples that were collected during 2007–2014 from 48 *P. knowlesi*–positive macaques had sufficient DNA for multilocus genotyping. Most were from long-tailed macaques, sampled from Selangor (n = 17), Perak (n = 6), and Negeri Sembilan (n = 15) in peninsular Malaysia and from Balingian (n = 1), Limbang (n = 2), Miri (n = 2), and Sarikei (n = 2) in Sarawak; pig-tailed macaque samples were from Limbang (n = 1) and Kapit (n = 2) in Sarawak. We performed the sampling according to the protocols of the Department of Wildlife and National Parks in Malaysia. We included DNA of *P. knowlesi* strain Nuri (kindly provided by Clemens Kocken at the Biomedical Primate Research Centre, the Netherlands) in the genotyping as a control (*17*).

### Microsatellite Genotyping of New Samples

We genotyped each of the *P. knowlesi*–positive DNA samples at 10 microsatellite loci (NC03_2, CD05_06, CD08_61, NC0AU: 9_1, NC10_1, CD11_157, NC12_2, NC12_4, CD13_61, CD13_107) using hemi-nested PCR assays specific for *P. knowlesi*, as described previously (*9*). We analyzed fluorescent dye-labeled PCR products by using capillary electrophoresis on the Genetic Analyzer 3730 (Applied Biosystems, Cheshire, UK), with GeneScan 500 LIZ internal size standards, following which we scored alleles and peak heights with GeneMapper version 4.0 software (Applied Biosystems).

The genotypic multiplicity of infection (MOI) was defined as the maximum number of alleles detected at any individual locus. Electrophoretic peak heights above 200 fluorescent units of the expected molecular sizes were scored as alleles, and secondary peaks within an infection sample were scored if they had a height of at least 25% relative to the predominant allele. We determined the multilocus genotype profile of each infection, and allele frequency counts for population samples, by counting the predominant allele at each locus within each infection.

### Analysis of Microsatellite Genotypes from Previous Data

We retrieved whole genome sequence data of *P. knowlesi* samples from the European Nucleotide Archive (http://www.ebi.ac.uk/ena), and we obtained the reference genome sequence of strain H from GeneDB (http://www.genedb.org/Homepage/Pknowlesi). Most of the parasite genome short-read Illumina sequences available are from patients sampled in Malaysian Borneo (*12*,*16*), but a few are from older laboratory lines that originated from peninsular Malaysia, as well as 1 supposedly from the Philippines (*16*). Although genome sequences indicate some historical mislabeling or contamination of the laboratory lines, meaning that individual identities are in question, it is clear that most are from peninsular Malaysia (*16*). We aligned the raw short reads to *P. knowlesi* genome strain H by using the BWA-MEM alignment tool with default parameters (https://arxiv.org/pdf/1303.3997.pdf). We identified lists of indels using the SAMTools and VCFtools software (*18*,*19*) with the following parameters, described elsewhere (*16*): *mpileup –B –Q 23 –d 2000 –C 50 -ugf; varFilter –d 10 –D 2000*. Using ARTEMIS software (*20*), we determined the putative microsatellite allele size by inspecting the indels within the location of the PCR primers used for the second amplification PCR. We assessed the quality of the mapping within the microsatellite allele regions with the minimum depth of short-read coverage at 30-fold.

### Analyses of *P. knowlesi* Population Genetic Substructure

We evaluated population genetic structure by Bayesian clustering inference using STRUCTURE version 2.3.4 software (*21*), on samples for which there were no missing data at any locus. First, to allocate the probable ancestral assignment of a genotype into 1 or more *K* clusters, we set the parameters for the admixture model on the basis of correlated allele frequency, without providing the sample source information. However, the sensitivity for population structure analysis can be improved by providing population information, in which an algorithm assumes that the probability of an individual being part of a population varies among locations or sources of origins (*22*). For the second test, we set the parameter to LOCPRIOR. This parameter is informative when population structure signals are weak because of a close relationship between populations. We performed both LOCPRIOR and non-LOCPRIOR parameters in STRUCTURE runs separately with a burn-in period of 50,000 followed by 100,000 Markov chains (MCMC iterations). The simulations were replicated 20 times for *K* values ranging from 1 to 10. The optimal *K* value was calculated based on Evanno’s method of *ΔK* statistics implemented in the STRUCTURE HARVESTER webpage interface (*23*,*24*). For the optimum *K*, we aligned the 20-replicate runs at 10,000 permutations to determine the consensus of cluster scores using CLUMPP version 1.1.2 (*25*).

To evaluate population structure independently, we performed principal coordinate analysis (PCoA) using the GenAlEx package version 6 implemented in Microsoft Excel (*26*). We first generated a genetic distance matrix using the multilocus microsatellite dataset, and we plotted a 2-dimensional PCoA based on the first 2 highest eigenvalues. We calculated the *K*-means clusters using the first and second eigenvectors generated from the PCoA, and subsequently used them to assign each individual infection to the most probable cluster. In addition, we applied the discriminant analysis of principal component from the *adegenet* 2.0.0 packages in R to assess the population structure (*27*). In this procedure, we first transformed genotype data into 40 uncorrelated principal components, and then, using the discriminant function, we partitioned the variances into within-group and among-group components, while optimizing separations between groups.

We calculated pairwise differentiation (*F_ST_*) between different subpopulations of *P. knowlesi* by using FSTAT software version 2.9.3.2 (*28*). We estimated the mean allelic diversity across loci, measured as expected heterozygosity (*H_E_*), using FSTAT software. We assessed multilocus linkage disequilibrium with the standardized index of association (*I_A_^S^*), calculated by LIAN version 3.7 (*29*), with Monte Carlo simulation of 10,000 data permutations.

## Results

### Genotypic Diversity within *P. knowlesi* Infections

Of 182 *P. knowlesi* infections genotyped for this study (134 from humans, 45 from long-tailed macaques, and 3 from pig-tailed macaques), 166 (91.2%) yielded complete genotype data for the panel of 10 microsatellite loci, whereas the remainder were each genotyped for at least 7 of the loci (Table; Technical Appendix 1).

**Table:** Summary of *Plasmodium knowlesi* mixed-genotype infections in 134 human and 48 macaque hosts across Malaysia obtained using 10 microsatellite loci*

Host and site	Region	No. samples	No. isolates by no. genotypes detected				% Poly	Average MOI	MS_10_
			1	2	3	4			
Human									
Kapit	Sarawak	35	27	5	2	1	23	1.34	35
Betong	Sarawak	4	4	0	0	0	0	1.00	3
Lawas	Sarawak	15	7	7	0	1	53	1.67	14
Kudat	Sabah	20	13	6	1	0	35	1.40	20
Ranau	Sabah	25	13	10	2	0	48	1.56	25
Tenom	Sabah	22	11	7	3	1	50	1.73	22
Kelantan	Peninsular Malaysia	13	5	6	2	0	62	1.77	13
Total		134							132
Long-tailed macaque									
Balingian	Sarawak	1	0	1	0	0	100	2.00	1
Limbang	Sarawak	2	0	1	1	0	100	2.50	1
Miri	Sarawak	2	1	1	0	0	50	1.50	1
Sarikei	Sarawak	2	1	0	1	0	50	2.00	2
Selangor	Peninsular Malaysia	17	8	6	2	1	53	1.76	15
Perak	Peninsular Malaysia	6	1	3	2	0	83	2.17	5
Negeri Sembilan	Peninsular Malaysia	15	0	3	6	6	100	3.20	6
Total		45							31
Pig-tailed macaque									
Limbang	Sarawak	1	0	0	1	0	100	3.00	1
Kapit	Sarawak	2	1	0	1	0	50	2.00	2
Total		3							3
All		182							166

*All samples were successfully genotyped at ≥7 loci, and 166 samples had complete genotypes for all 10 microsatellite loci (MS_10_). MOI, multiplicity of infection; poly, polyclonal infections.

**Figure 2:**
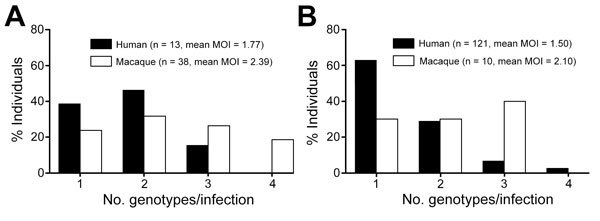
Multiplicity of infection (MOI) for *Plasmodium knowlesi* genotypes in 134 human and 48 macaque hosts across Malaysia. Means of MOI were higher in macaque hosts than in human hosts for both regions, but the values were not statistically significant for A) peninsular Malaysia (p = 0.25 by Fisher exact test) compared with B) Malaysian Borneo (p = 0.01).

Among the human cases, single genotype infections were common, and the average number of genotypes per infection (MOI) was less than 2 at all sites sampled. This was expected when these samples were collectively reanalyzed with *P. knowlesi* infections of humans and macaques across Malaysia from previous studies (Technical Appendix 2 Table 1). We found no notable difference in numbers of genotypes per infection in Malaysian Borneo and peninsular Malaysia (mean MOI values of 1.50 and 1.77, respectively; p = 0.14 by Fisher exact test). In contrast, multiple genotype infections were more common in macaques both in Malaysian Borneo (mean MOI = 2.10, p = 6.7 × 10^−3^) and peninsular Malaysia (mean MOI = 2.39, p = 9.8 × 10^−4^) (Table; Figure 2). We counted the predominant allele at each locus per infection for subsequent statistical analyses on population structure.

### Analysis of *P. knowlesi* Population Genetic Structure with New Samples

**Figure 3:**
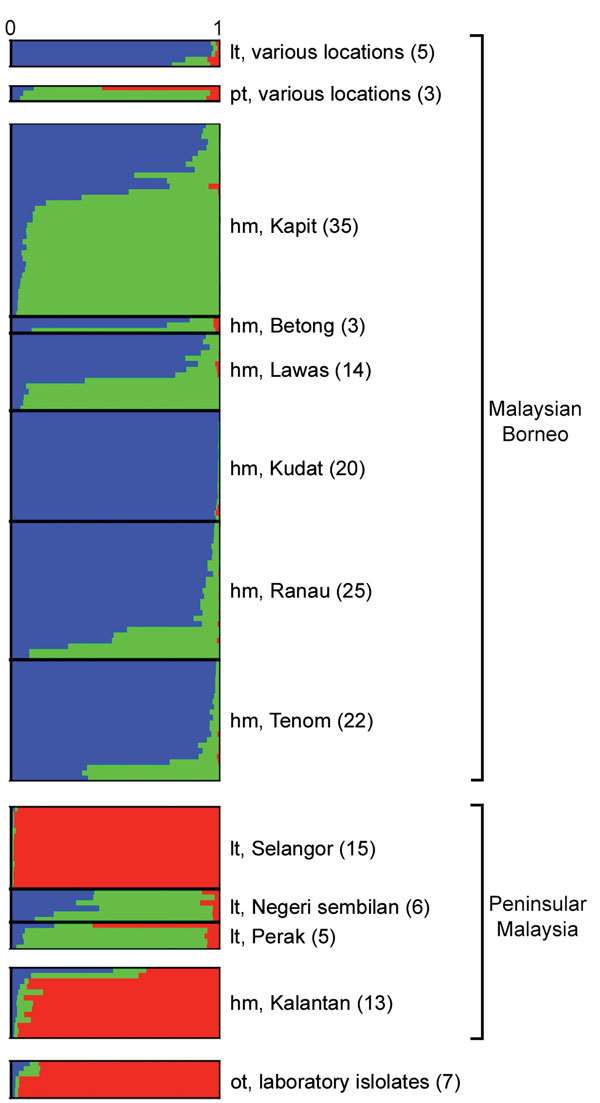
Subpopulation cluster assignments of individual *Plasmodium knowlesi* infections in human and macaque hosts across Malaysia and 7 laboratory isolates. The Bayesian-based STRUCTURE analysis with LOCPRIOR model (*22*) was applied on complete 10-microsatellite loci of 166 *P. knowlesi* infections and 7 laboratory isolates showing 3 subpopulation clusters (*K* = 3; *ΔK* = 37.72). Ancestral population clusters are referred to as cluster 1 (blue), cluster 2 (green), and cluster 3 (red). Numbers in parentheses indicate number of isolates. hm, human; lt, long-tailed macaque; pt, pig-tailed macaque; ot, various other sources.

Bayesian clustering analyses using 2 admixture models on the new sample of 166 infections with complete genotype data for the full panel of 10 microsatellite loci identified 3 subpopulation clusters (*K* = 3; Figure 3; Technical Appendix 2 Figures 1 and 2, panel A), hereafter referred to as clusters 1–3. Human infections in Malaysian Borneo were assigned to clusters 1 and 2, whereas long-tailed macaque infections were all in cluster 1 and pig-tailed macaque infections were mostly in cluster 2 (1 pig-tailed macaque infection was assigned as intermediate between clusters 2 and 3), confirming the existence of 2 major sympatric subpopulations in Malaysian Borneo, as reported previously (*9*,*12*,*16*).

Among the samples from peninsular Malaysia, those from human cases were all assigned to cluster 3, along with most of the infections from wild long-tailed macaques sampled in Kelantan, although long-tailed macaque infections from the other 2 sites had more intermediate cluster assignments, suggesting some ancestral affinity with cluster 2. All the laboratory isolates, originating many years ago mainly from peninsular Malaysia, were clearly assigned to cluster 3, consistent with results of a recent whole genome sequence analysis (*16*).

### Analysis of Population Genetic Structure Incorporating New and Previously Acquired Microsatellite Data

To further evaluate the population structure of *P. knowlesi*, we collated the dataset in this study with data from samples analyzed previously (*9*). This yielded a total of 758 *P. knowlesi* infections with the complete panel of 10 microsatellite loci genotyped. This total comprises 166 samples from the present study (Table), 556 previously genotyped samples, 29 samples that had undergone repeat genotyping for all 10 loci completed here (Technical Appendix 2 Figure 3), and 7 derived from Illumina short-read sequence data.

**Figure 4:**
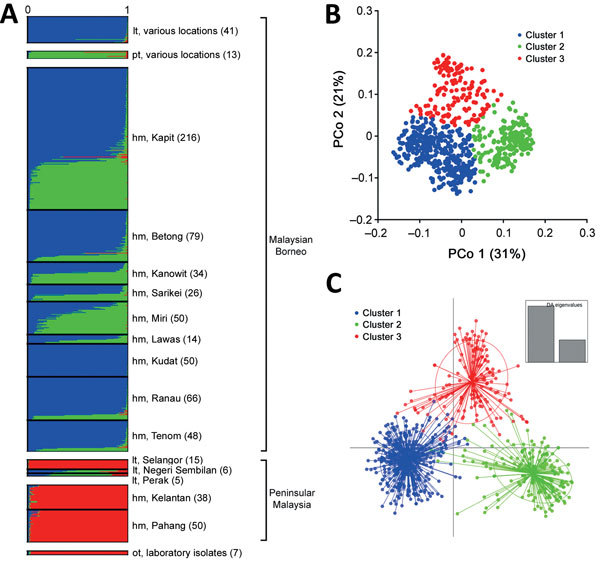
Population genetic structure of combined 751 *P. knowlesi* infections across Malaysia and 7 laboratory isolates. A) The inference of genetic clusters on complete 10-locus genotype dataset using the STRUCTURE analysis with LOCPRIOR model (*22*) showed 3 major subpopulation structures (*K* = 3, *ΔK* = 98.73), corresponding to those shown in Figure 3. Numbers in parentheses indicate number of isolates. B, C) Using a priori K = 3, individual genotypes were assigned to the most probable subpopulation clusters using independent genetic distance matrix inferred by the principal coordinate analysis (B) and discriminant analysis of principal component (DAPC) (C). In DAPC, clusters depicted as ellipses indicated the variance within the clusters and centered by *K*-means. hm, human; lt, long-tailed macaque; PCo, principal coordinate; pt, pig-tailed macaque.

The admixture STRUCTURE analysis without the LOCPRIOR model identified 2 subpopulation clusters (*K* = 2; Technical Appendix 2 Figure 2, panel B, and 4). This was consistent with a previous analysis showing that human cases in the Malaysian Borneo group fell into 2 different genotype clusters, which are also respectively seen in long-tailed and pig-tailed macaque infections, although the current analysis assigned samples from peninsular Malaysia to cluster 2 (previously, they had been grouped into cluster 1). However, incorporation of the LOCPRIOR model showed 3 subpopulation clusters (*K* = 3; Figure 4, panel A; Technical Appendix 2 Figure 4), with most of the isolates from peninsular Malaysia belonging to cluster 3, as also seen with the analysis based solely on the new samples. Overall, this confirms that human *P. knowlesi* infections in Malaysian Borneo are divided into 2 different genetic subpopulations that are associated with different macaque reservoir host species, whereas human infections in peninsular Malaysia belong to a third subpopulation that is also seen in long-tailed macaques at 1 of the sites in peninsular Malaysia.

### Robustness and Divergence of Subpopulation Clusters

Using an a priori designation of 3 subpopulation clusters (*K =* 3), we independently assigned all 758 infections into clusters using PCoA (Figure 4, panel B) and discriminant analysis (Figure 4, panel C), and compared the results with those derived from the STRUCTURE analysis (Figure 4, panel A). These showed highly concordant results (Technical Appendix 1). PCoA indicated that infections in humans were strongly associated with infections in local macaque reservoir hosts for both Malaysian Borneo and peninsular Malaysia (online Technical Appendix 2 Figure 5). Discriminant analysis also showed clear clustering, with only minimal overlap among the inertia ellipses for the 3 major clusters.

To test the consistency and robustness of cluster assignment for all 758 infections, across the different methods used (Bayesian analysis using STRUCTURE, principal coordinates analysis, and discriminant analysis), we assessed a consensus for each individual (Technical Appendix 1; Technical Appendix 2 Table 2). A large majority (86.4%) of infections were assigned into the same cluster by all 3 methods (cluster 1, n = 384; cluster 2, n = 175; cluster 3, n = 96). Most of the remainder (12.9% of the total) had an agreed assignment for 2 of the methods (cluster 1, n = 65; cluster 2, n = 16; cluster 3, n = 17), whereas only 5 (0.7%) showed no agreement across the methods. Omitting the few infections that did not show agreement for 2 or more methods yielded a dataset of 753 *P. knowlesi* infections that grouped into 3 major subpopulation clusters (cluster 1, n = 449; cluster 2, n = 191; cluster 3, n = 113; Technical Appendix 2 Table 3). We estimated values of allelic diversity (*H_E_*) between 0.51 and 0.83 among different sites at each of the subpopulation clusters (Technical Appendix 2 Table 4), and we observed similar patterns even without separating the infections by subpopulation cluster assignments (Technical Appendix 2 Table 5). The index of multilocus linkage disequilibrium yielded various degrees (*I_A_^S^* range from −0.007 to 0.305), with loss of significance at majority of the sites among the 3-subpopulation clusters (Technical Appendix 2 Table 4). However, the degree of significance increased when all infections were not assigned into subpopulation clusters (*I_A_^S^* range from −0.002 to 0.242 with p<0.01 at most sites; Technical Appendix 2 Table 5). Analyses of allele frequencies across all 10 microsatellite loci confirmed strong genetic differentiation among these clusters (*F_ST_* = 0.184 between clusters 1 and 2; *F_ST_* = 0.152 between clusters 1 and 3; *F_ST_* = 0.201 between clusters 2 and 3; p<3.3 × 10^−4^ for each comparison using 3,000 randomized permutations). This indicates deep divergence among the 3 major parasite subpopulations that infect humans, 2 of which are sympatric and predominantly associated with different reservoir hosts (long-tailed and pig-tailed macaques in Malaysian Borneo), and 1 of which is allopatric in a different geographic region (peninsular Malaysia).

## Discussion

Three major subpopulations of *P. knowlesi* have been demonstrated in natural human infections in Malaysia. These subpopulations show profound divergence, with pairwise *F_ST_* values of ≈0.2, suggesting minimal or no current gene flow between parasites in Malaysian Borneo and peninsular Malaysia, nor between parasites in long-tailed and pig-tailed macaque hosts within Malaysian Borneo.

The existence of 3 divergent clusters was initially indicated from whole genome sequence-based single nucleotide polymorphism analysis of *P. knowlesi* clinical isolates and laboratory lines (*16*). Whereas 2 of the clusters of genome sequences (clusters 1 and 2) had been seen in clinical infections in Malaysian Borneo, the third (cluster 3) was seen only in old laboratory lines that were originally isolated mostly from peninsular Malaysia. Using microsatellite scoring obtained from genome sequences and combined with genotyping of infections from humans and macaques in the current study, we confirmed that the cluster 3 subpopulation is widespread in peninsular Malaysia. Furthermore, it is divergent from clusters 1 and 2, which account for all infections in Malaysian Borneo and apparently a minority of wild macaque infections in peninsular Malaysia. With smaller numbers of samples, recent studies on sequence diversity in genes encoding the normocyte binding protein (*Pknbpxa*) (*10*) and the Duffy binding protein (*PkDBP*) (*30*), as well as the 18S rRNA gene and the mitochondrial *Cox1* gene, have suggested that parasites in peninsular Malaysia had probably diverged from those in Malaysian Borneo.

It is likely that allopatric divergence occurred as a result of the ocean barrier between Borneo and mainland Southeast Asia, established at the end of the last ice age ≈13,000 years ago, which prevents the movement of wild macaque reservoir hosts (*31*). However, one of the old laboratory lines that was recently sequenced is labeled as having originally been isolated from a long-tailed macaque in “Philippines,” and this sequence is clearly assigned to cluster 3 along with the parasites from peninsular Malaysia (*16*), although the islands of the Philippines have never been connected to peninsular Malaysia or any other part of mainland Southeast Asia (*32*). Unless there was a historical mislabeling or previous mixup of parasite material, this finding suggests that wider sampling of *P. knowlesi* in wild macaques will give a more complete understanding of divergence within this zoonotic parasite species (*31*,*33*–*35*). Similarly, the observation that a minority of *P. knowlesi* parasites in long-tailed macaques from peninsular Malaysia are assigned to cluster 2, which has otherwise been seen only in samples from Malaysian Borneo, indicates that additional sampling of macaques from different areas may uncover more features of the parasite population structure.

The sympatric differentiation between cluster 1 and cluster 2 parasites in Malaysian Borneo supports the idea that parasite subpopulations are transmitted independently in long-tailed and pig-tailed macaque populations (*36*,*37*). Although pig-tailed macaques occur mostly in forested areas, long-tailed macaques have a broader habitat range in both forested and nonforested areas (*38*). Because of the absence of parasite samples from pig-tailed macaques in peninsular Malaysia, it is unknown whether there is divergence in *P. knowlesi* between the different macaque host species in this region.

Analysis of genome sequences to derive the frequency distribution of single-nucleotide polymorphism alleles indicates that the cluster 1 subpopulation of *P. knowlesi* has undergone long-term population growth (*16*). It is unknown whether parasites of cluster 2 and cluster 3 subpopulations have a similar demographic history, but genome sequencing of more samples within these subpopulations should be able to address this in the future.

The observation that most infections in all macaque populations are polyclonal, whereas most human cases contain single parasite genotypes, probably reflects a higher intensity of transmission among macaques than from macaques to humans (*9*). It is not yet known whether there are any substantial differences in the clinical course of infections caused by the 3 major subpopulations of *P. knowlesi*; this question should be investigated in a manner that accounts for any confounding variables between different study sites. In any case, recognition of these divergent subpopulations provides a more accurate basis on which to understand and potentially control the transmission of this zoonosis. Furthermore, obtaining whole-genome sequence data from more clinical samples belonging to each of the 3 major types should enable a more thorough investigation of the genomic divergence, and identify loci at which there are signals of recent adaptation that may relate to differences in virulence or transmission.

Technical Appendix 1 Additional datasets regarding *Plasmodium knowlesi* genotypes in humans and macaques studied in Malaysian Borneo and peninsular Malaysia.

Technical Appendix 2 Additional information regarding *Plasmodium knowlesi* genotypes in humans and macaques studied in Malaysian Borneo and peninsular Malaysia.
