# Supplementary material for: Three Divergent Subpopulations of the Malaria Parasite Plasmodium knowlesi
Source: Emerg Infect Dis. 2017 Apr;23(4):616–24. doi: 10.3201/eid2304.161738 (PMC5367419; doi:10.3201/eid2304.161738)
Supplement: Technical Appendix 2 — Additional information regarding Plasmodium knowlesi genotypes in humans and macaques studied in Malaysian Borneo and peninsular Malaysia. [file 16-1738-Techapp-s2.pdf]

Article DOI: http://dx.doi.org/10.3201/eid2304.161738

Three Divergent Subpopulations of the Malaria Parasite *Plasmodium knowlesi*

Technical Appendix 2

Technical Appendix 2 Table 1. Summary of *P. knowlesi* mixed genotype infections in 683 humans and 94 macaques across Malaysia obtained using 10 microsatellite loci*

			No. isolates with the following no. genotypes detected						
Host and Site	Region	N	1	2	3	4	5	%poly	MOI
Human									
Kapit	Sarawak	220	149	62	5	4	0	32	1.38
Betong	Sarawak	81	57	22	2	0	0	30	1.32
Kanowit	Sarawak	34	27	7	0	0	0	21	1.21
Sarikei	Sarawak	26	21	5	0	0	0	19	1.19
Miri	Sarawak	50	43	7	0	0	0	14	1.14
Lawas	Sarawak	15	7	7	0	1	0	53	1.67
Kudat	Sabah	50	32	15	3	0	0	36	1.42
Ranau	Sabah	66	31	31	4	0	0	53	1.59
Tenom	Sabah	48	27	17	3	1	0	44	1.54
Kelantan	Peninsular Malaysia	43	24	17	2	0	0	44	1.49
Pahang	Peninsular Malaysia	50	31	15	3	1	0	38	1.48
Long-tailed macaque									
Kapit	Sarawak	36	4	12	11	7	2	89	2.75
Balingian	Sarawak	1	0	1	0	0	0	100	2.00
Limbang	Sarawak	2	0	1	1	0	0	100	2.50
Miri	Sarawak	2	1	1	0	0	0	50	1.50
Sarikei	Sarawak	2	1	0	1	0	0	50	2.00
Selangor	Peninsular Malaysia	17	8	6	2	1	0	53	1.76
Perak	Peninsular Malaysia	6	1	3	2	0	0	83	2.17
Negeri Sembilan	Peninsular Malaysia	15	0	3	6	6	0	100	3.20
Pig-tailed macaque									
Kapit	Sarawak	12	1	6	3	1	1	92	2.58
Limbang	Sarawak	1	0	0	1	0	0	100	3.00

*All new and old samples (N) were genotyped at ≥7 loci. The percentage of polyclonal infections (%poly) and average genotypic multiplicity of infections (MOI) are shown.

Technical Appendix 2 Table 2. Assignment of combined 753 *P. knowlesi* genotypes into 3 subpopulation clusters determined by a minimum of 2 out of 3 assignment methods*

P. knowlesi population	Subpopulation cluster	DAPC ∧ PCoA ∧ LOC	DAPC ∧ PCoA	DAPC ∧ LOC	PCoA ∧ LOC	Total isolate
LT-various locations in Sarawak	Cluster 1	32	0	4	3	39
	Cluster 2	1	0	0	0	1
	Cluster 3	0	0	0	0	0
PT-various locations in Sarawak	Cluster 1	0	0	0	0	0
	Cluster 2	12	0	0	1	13
	Cluster 3	0	0	0	0	0
Hm-Kapit	Cluster 1	114	0	20	4	138
	Cluster 2	70	1	1	2	74
	Cluster 3	0	0	1	0	1
Hm-Betong	Cluster 1	57	0	10	0	67
	Cluster 2	10	2	0	0	12
	Cluster 3	0	0	0	0	0
Hm-Kanowit	Cluster 1	13	0	3	0	16
	Cluster 2	18	0	0	0	18
	Cluster 3	0	0	0	0	0
Hm-Sarikei	Cluster 1	14	0	0	1	15
	Cluster 2	11	0	0	0	11
	Cluster 3	0	0	0	0	0
Hm-Miri	Cluster 1	16	1	0	0	17
	Cluster 2	28	0	5	0	33
	Cluster 3	0	0	0	0	0
Hm-Lawas	Cluster 1	8	0	0	0	8
	Cluster 2	6	0	0	0	6
	Cluster 3	0	0	0	0	0
Hm-Kudat	Cluster 1	44	0	6	0	50
	Cluster 2	0	0	0	0	0
	Cluster 3	0	0	0	0	0
Hm-Ranau	Cluster 1	49	0	6	2	57
	Cluster 2	8	1	0	0	9
	Cluster 3	0	0	0	0	0
Hm-Tenom	Cluster 1	37	0	5	0	42
	Cluster 2	5	0	1	0	6
	Cluster 3	0	0	0	0	0
LT-Selangor	Cluster 1	0	0	0	0	0
	Cluster 2	0	0	0	0	0
	Cluster 3	14	0	1	0	15
LT-Negeri Sembilan	Cluster 1	0	0	0	0	0
	Cluster 2	2	0	1	1	4
	Cluster 3	0	1	0	0	1
LT-Perak	Cluster 1	0	0	0	0	0
	Cluster 2	4	0	0	0	4
	Cluster 3	1	0	0	0	1
Hm-Kelantan	Cluster 1	0	0	0	0	0
	Cluster 2	0	0	0	0	0
	Cluster 3	33	0	5	0	38
Hm-Pahang	Cluster 1	0	0	0	0	0
	Cluster 2	0	0	0	0	0
	Cluster 3	43	0	5	2	50
Laboratory isolates	Cluster 1	0	0	0	0	0
	Cluster 2	0	0	0	0	0
	Cluster 3	5	0	2	0	7

*Five genotypes from long-tailed macaques in Sarawak (n = 1), humans in Kapit (n = 3), and long-tailed macaques in Negeri Sembilan (n = 1) showed inconsistency in cluster assignment methods are not shown in this table. DAPC, discriminant analysis of principal component; Hm – human; LOC, STRUCTURE analysis with LOCPRIOR model; LT, long-tailed macaque; PCoA, principal coordinate analysis based on genetic distance matrix; PT, pig-tailed macaque.

Technical Appendix 2 Table 3. Summary of subpopulation cluster assignment on combined 758 *P. knowlesi* genotypes according to host and geographic origins

Subpopulation	Malaysian Borneo			Peninsular Malaysia		Laboratory	Total
cluster	LT*	PT	Hm	LT	Hm	isolate	isolate
Cluster 1	39	0	410	0	0	0	449
Cluster 2	1	13	169	8	0	0	191
Cluster 3	0	0	1	17	88	7	113
Unassigned	1	0	3	1	0	0	5

*Hm, human; LT, long-tailed macaque; PT, pig-tailed macaque.

Technical Appendix 2 Table 4. Multilocus linkage disequilibrium (standardized index of association) and genetic diversity (expected heterozygosity) of *P. knowlesi* infections for each of the 3 subpopulation clusters separately at different geographic sites*

Site	Region	Host	N	I_A^S	p value	H_E (±SE)
Cluster 1						
Kapit	Sarawak	Long-tailed macaque	34	0.026	<0.01	0.70 (0.05)
Kapit	Sarawak	Human	138	0.003	0.19	0.70 (0.06)
Betong	Sarawak	Human	67	0.005	0.25	0.63 (0.08)
Kanowit	Sarawak	Human	16	0.007	0.33	0.67 (0.05)
Sarikei	Sarawak	Human	15	0.003	0.42	0.64 (0.08)
Miri	Sarawak	Human	17	0.038	0.01	0.67 (0.09)
Lawas	Sarawak	Human	8	0.026	0.27	0.71 (0.09)
Kudat	Sabah	Human	50	−0.003	0.65	0.68 (0.07)
Ranau	Sabah	Human	57	0.011	0.02	0.71 (0.06)
Tenom	Sabah	Human	42	0.006	0.22	0.70 (0.06)
Cluster 2						
Kapit	Sarawak	Pig-tailed macaque	12	0.004	0.38	0.58 (0.09)
Kapit	Sarawak	Human	74	0.003	0.27	0.59 (0.10)
Betong	Sarawak	Human	12	0.083	<0.01	0.62 (0.11)
Kanowit	Sarawak	Human	18	−0.009	0.76	0.62 (0.08)
Sarikei	Sarawak	Human	11	0.047	0.04	0.59 (0.10)
Miri	Sarawak	Human	33	0.013	0.10	0.63 (0.08)
Lawas	Sarawak	Human	6	0.076	0.08	0.51 (0.08)
Ranau	Sabah	Human	9	0.099	<0.01	0.64 (0.10)
Tenom	Sabah	Human	6	−0.007	0.66	0.64 (0.08)
Negeri Sembilan	Peninsular Malaysia	Long-tailed macaque	4	0.085	0.26	0.83 (0.06)
Perak	Peninsular Malaysia	Long-tailed macaque	4	0.305	<0.01	0.58 (0.08)
Cluster 3						
Selangor	Peninsular Malaysia	Long-tailed macaque	15	0.044	0.01	0.62 (0.03)
Kelantan	Peninsular Malaysia	Human	38	0.020	0.03	0.71 (0.04)
Pahang	Peninsular Malaysia	Human	50	−0.001	0.56	0.72 (0.04)

All new and old samples (N) were genotyped at complete 10 microsatellite loci. Sites with N<4 were not included in the analyses.
H_E, expected heterozygosity; I_A^S, standardized index of association.

Technical Appendix 2 Table 5. Multilocus linkage disequilibrium (standardized index of association) and genetic diversity (expected heterozygosity) of *P. knowlesi* infections sampled from different host species at each site, without separating by subpopulation cluster assignment*

Site	Region	Host	N	I_A^S	p value	H_E (±SE)
Kapit	Sarawak	Long-tailed macaque	36	0.023	<0.01	0.71 (0.05)
Kapit	Sarawak	Pig-tailed macaque	12	0.004	0.40	0.58 (0.09)
Kapit	Sarawak	Human	216	0.025	<0.01	0.74 (0.04)
Betong	Sarawak	Human	79	0.020	<0.01	0.67 (0.08)
Kanowit	Sarawak	Human	34	0.024	<0.01	0.72 (0.05)
Sarikei	Sarawak	Human	26	0.040	<0.01	0.71 (0.08)
Miri	Sarawak	Human	50	0.031	<0.01	0.60 (0.04)
Lawas	Sarawak	Human	14	0.050	0.01	0.72 (0.05)
Kudat	Sabah	Human	50	–0.003	0.64	0.68 (0.07)
Ranau	Sabah	Human	66	0.026	<0.01	0.75 (0.05)
Tenom	Sabah	Human	48	0.009	0.10	0.73 (0.04)
Selangor	Peninsular Malaysia	Long-tailed macaque	15	0.044	0.01	0.62 (0.08)
Negeri Sembilan	Peninsular Malaysia	Long-tailed macaque	6	0.028	0.24	0.78 (0.06)
Perak	Peninsular Malaysia	Long-tailed macaque	5	0.242	<0.01	0.69 (0.06)
Kelantan	Peninsular Malaysia	Human	38	0.020	0.03	0.71 (0.04)
Pahang	Peninsular Malaysia	Human	50	–0.002	0.56	0.72 (0.04)

*All new and old samples (N) were genotyped at complete 10-microsatellite loci. Sites with N<4 were not included in the analyses. H_E, expected heterozygosity; I_A^S, standardized index of association.

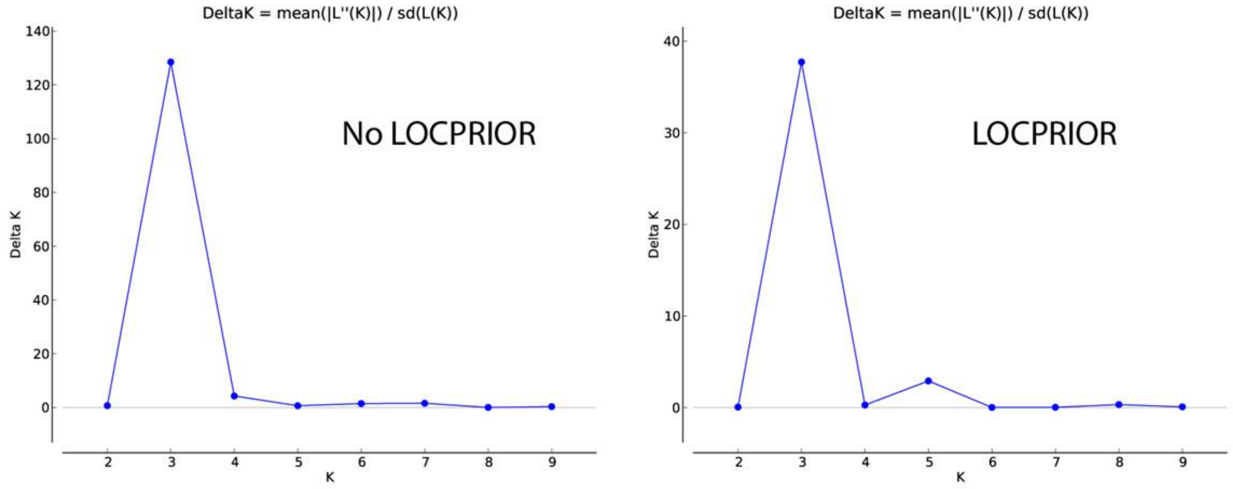

Technical Appendix 2 Figure 1. STRUCTURE analysis on 166 *P. knowlesi* infections across Malaysia and seven laboratory isolates obtained by using 10 microsatellite loci. Using both admixture with and without the LOCPRIOR models, we estimated 3 subpopulation clusters by Evanno's method (K = 3; ΔK = 128.51 for the non-LOCPRIOR model and ΔK = 37.72 for the LOCPRIOR model).

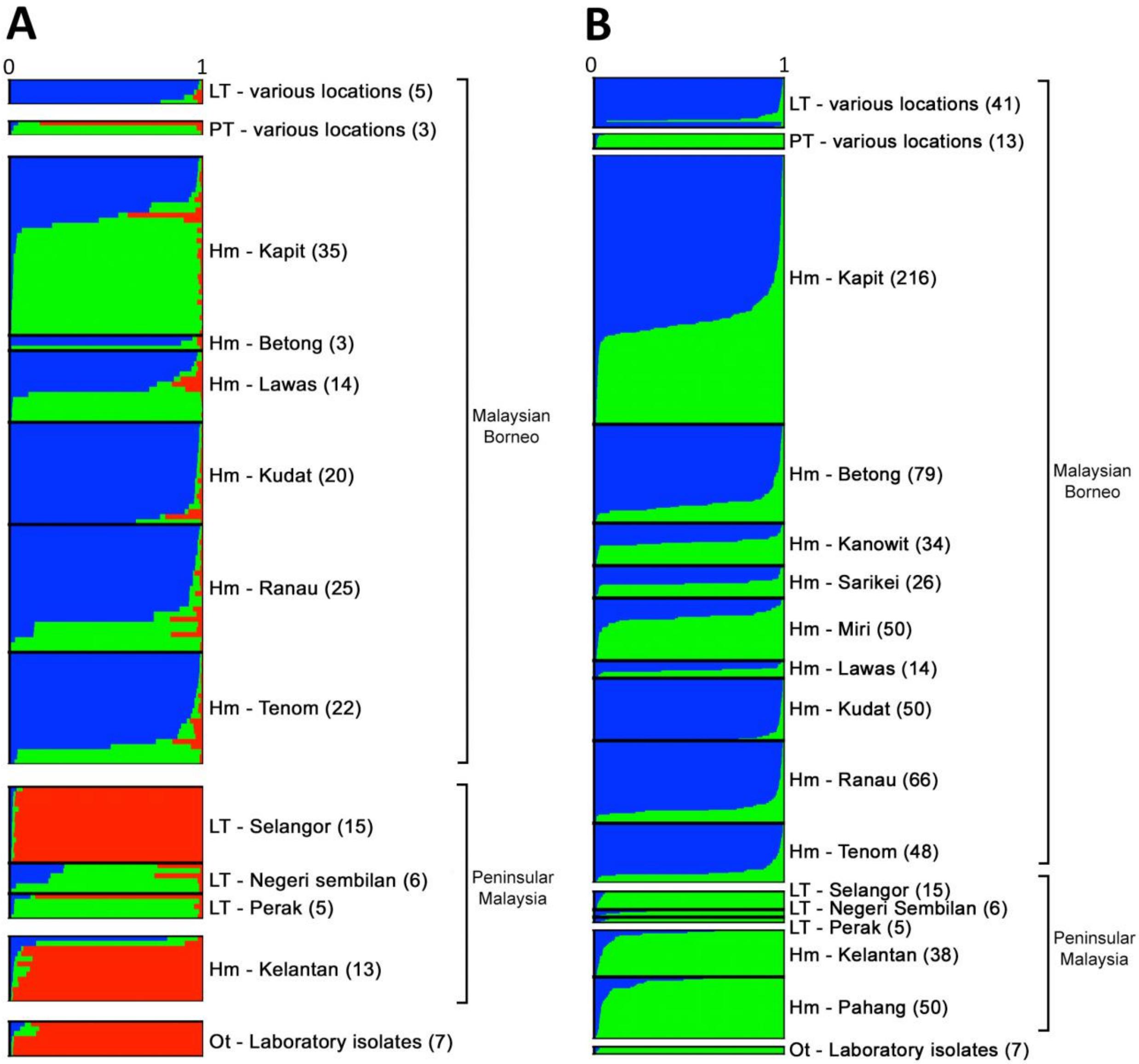

Technical Appendix 2 Figure 2. Cluster assignment of individual *P. knowlesi* genotypes obtained by using the Bayesian-based STRUCTURE algorithm without the LOCPRIOR model. For both analyses, complete 10-locus microsatellite genotyping on (A) 166 *P. knowlesi* infections in the present study and 7 laboratory isolates showed $K = 3$ with $\Delta K = 128.51$, and (B) a combined 758 *P. knowlesi* genotypes showed $K = 2$ with $\Delta K = 255.50$. Ancestral population clusters are referred to as cluster 1 (blue), cluster 2 (green), and cluster 3 (red). Hm, human; LT, long-tailed macaque; PT, pig-tailed macaque; Ot, various sources.

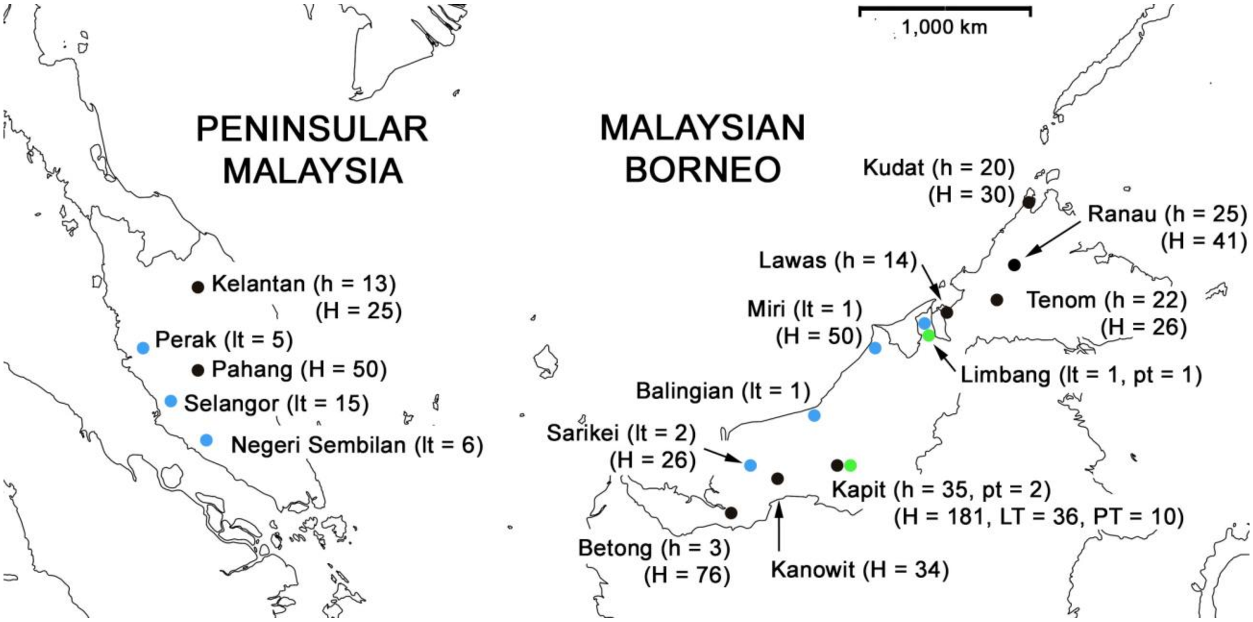

Technical Appendix 2 Figure 3. Total samples of *P. knowlesi* infections derived from 671 humans and 80 macaques across Malaysia with complete 10-microsatellite loci. Samples obtained from humans are marked with black dots and labeled with “h” for new samples and “H” for old samples; samples from long-tailed macaques are marked with blue dots and labeled with “lt” for new samples and “LT” for old samples; and samples from pig-tailed macaques are marked with green dots and labeled with “pt” for new samples and “PT” for old samples.

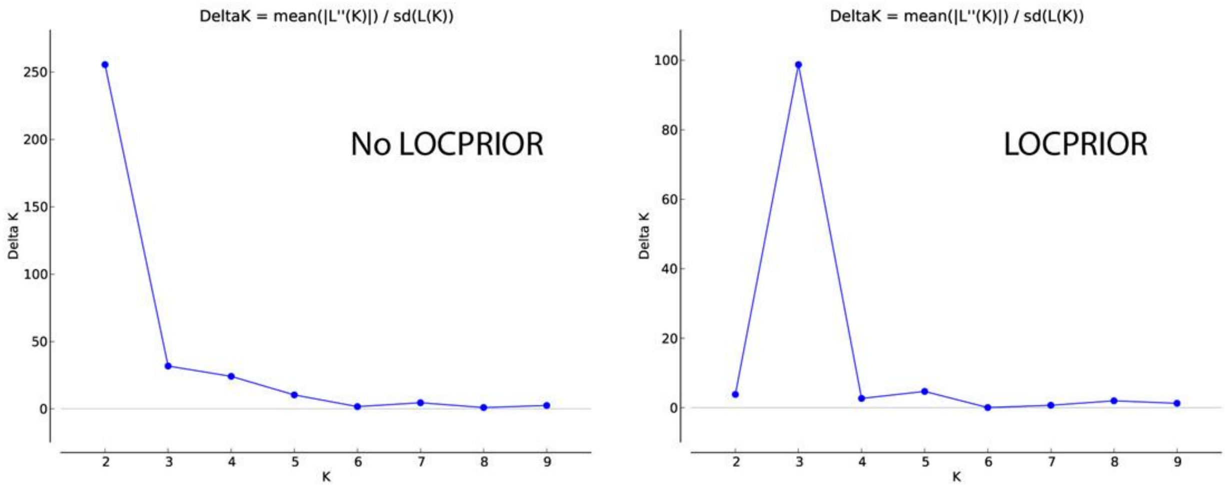

Technical Appendix 2 Figure 4. STRUCTURE analysis on 758 *P. knowlesi* genotypes obtained using 10 microsatellite loci. Estimated by Evanno’s method, analysis of admixture without the LOCPRIOR model showed 2 subpopulation clusters (K = 2, ΔK = 255.50), whereas admixture with LOCPRIOR model showed 3 subpopulation clusters (K = 3, ΔK = 98.73).

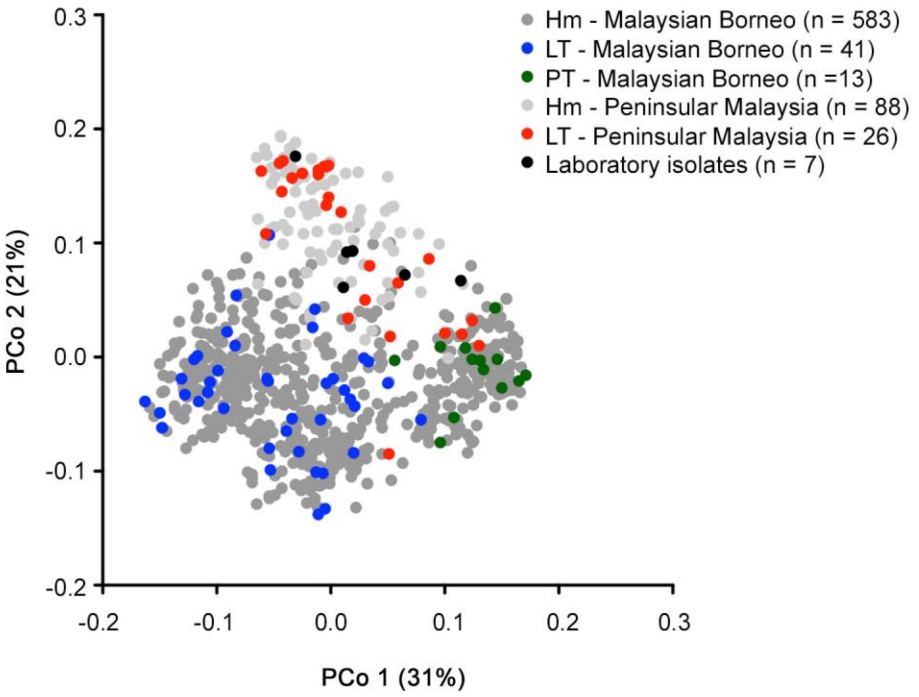

Technical Appendix 2 Figure 5. Principal coordinate analysis deduced from genetic distance matrix of 10 microsatellite loci in 751 *P. knowlesi* infections across Malaysia and 7 laboratory isolates. Hm, human; LT, long-tailed macaque; PT, pig-tailed macaque.
